# Tooth and Bone Parameters in the Assessment of the Chronological Age of Children and Adolescents Using Neural Modelling Methods

**DOI:** 10.3390/s21186008

**Published:** 2021-09-08

**Authors:** Katarzyna Zaborowicz, Barbara Biedziak, Aneta Olszewska, Maciej Zaborowicz

**Affiliations:** 1 Department of Craniofacial Anomalies, Poznań University of Medical Sciences, Collegium Maius, Fredry 10, 61-701 Poznań, Poland; biedziak@ump.edu.pl (B.B.); anetaol@ump.edu.pl (A.O.); 2 Department of Biosystems Engineering, Poznan University of Life Sciences, Wojska Polskiego 50, 60-637 Poznań, Poland

**Keywords:** chronological age, dental age, age assessment, digital pantomography, digital image analysis, neural network image analysis, neural modeling

## Abstract

The analog methods used in the clinical assessment of the patient’s chronological age are subjective and characterized by low accuracy. When using those methods, there is a noticeable discrepancy between the chronological age and the age estimated based on relevant scientific studies. Innovations in the field of information technology are increasingly used in medicine, with particular emphasis on artificial intelligence methods. The paper presents research aimed at developing a new, effective methodology for the assessment of the chronological age using modern IT methods. In this paper, a study was conducted to determine the features of pantomographic images that support the determination of metric age, and neural models were produced to support the process of identifying the age of children and adolescents. The whole conducted work was a new methodology of metric age assessment. The result of the conducted study is a set of 21 original indicators necessary for the assessment of the chronological age with the use of computer image analysis and neural modelling, as well as three non-linear models of radial basis function networks (RBF), whose accuracy ranges from 96 to 99%. The result of the research are three neural models that determine the chronological age.

## 1. Introduction

The first part of this paper reviews the literature beginning with issues related to dental and metric age assessment. The commonly used methods are referred to and their advantages and disadvantages indicated. The following paragraphs present the possibility of using computer and neuronal image analysis methods in medicine and indicate the area of using artificial intelligence methods in dentistry. Then the material and research methods are described, a set of 21 original indicators is presented, which were the basis of the research and further analysis. Moreover, the three best generated network models that accomplish the task of metric age assessment are presented. The whole was crowned with a discussion and conclusion.

Metric age assessment is particularly useful for doctors to plan and evaluate the results of treatment, in anthropology and forensic medicine to determine the metric age of human remains, and to determine the age of children in the case of international adoptions or in individuals illegally staying in a given country.

The analog methods used in the clinical assessment of the patient’s chronological age, based on the development of his or her dentition, are subjective and characterized by low accuracy. These methods are not reliable because there are noticeable discrepancies between the chronological age and the predicted age determined using relevant scientifically developed tables, charts, and atlases [1,2,3,4,5,6,7,8,9,10,11,12,13,14,15,16,17,18]. The variations in determining the chronological age may be significant [1,2] as they can be as high as 36 months [3]. Moreover, the methods currently used are time-consuming: the dentist has to assess the stage of development of the tooth buds of the whole or half of the dentition himself.

Methods of assessing the dental age are divided into clinical methods, comparing the time of eruption of specific tooth groups and pantomographic methods, assessing the mineralization process of tooth buds.

Clinical methods, which assess the presence of tooth groups or individual teeth in a patient, allow to determine the dental age during a routine dental check-up. These methods are considered by dentists to be easy to use, relatively fast and non-invasive. However, the main drawbacks of these methods include inaccuracy, due to the difficulty of determining whether a tooth in the process of eruption should be counted among teeth that have already reached the occlusal plane, and the presence of factors disturbing the time of eruption [4].

Clinical methods of assessing dental age determine the age on the basis of tables and growth charts, from which the average time of eruption is determined. The most frequently quoted methods come from 1928 (Matiegka and Lukasova method) and 1968 (Charzewski and Panek method) [5]. Due to the phenomenon of acceleration, i.e., accelerated tooth eruption in the population, these methods are nowadays rarely used for precise assessment of the chronological age. They are mainly used for the initial assessment of the patient’s age and the detection of possible dental abnormalities.

Pantomographic methods of dental age assessment are methods based on the assessment of mineralization of tooth buds. They are used for more precise assessment of dental age than that using tables and growth charts. Many systems have been developed which differ in the number and type of evaluated teeth and the number of individual stages of teeth development [6,7,8,9].

Most methods of assessing dental age are based on the teeth of the mandible. This is due to the fact that the tooth buds develop earlier in the mandible. The exceptions are the third molars, which are formed first in the maxilla [9,10]. Additionally, the presence of maxillary sinuses on pantomographic images makes it difficult to assess the development of tooth buds in the superior arch.

Among the many methods of assessing the dental age [6,7,8,9,11,12,13,14], the Demirjian method is the most popular. It is used to assess the shape of the cavity and canal, as well as the degree of enamel and dentin formation. Due to the magnification of 3–10% occurring in the pantomographic picture, the tooth size is not analyzed. In this method, 7 permanent teeth on the left side of mandible are evaluated. There are eight stages of development obtained directly from the tables—separate for the male sex, separate for the female sex [15,16].

Another commonly used method is the Schour and Massler method, sometimes referred to as the atlas method, in which, based on the developmental pattern of deciduous and permanent teeth, 21 stages are distinguished and all teeth are assessed simultaneously [12].

In 1944, the Ubelaker method was developed, which, similarly to the Schour and Massler method, distinguishes 21 stages of development and is currently used worldwide in patients between 5 months and 35 years of age [17].

The latest analog method of assessing dental age is the London Atlas, which was developed in 2009 and presents 31 stages of development and eruption of teeth from 30 weeks in utero to 23 years of age. The data for the Atlas were obtained from 72 prenatal and 104 postnatal skeletal remains of children and young adults of known age-at-death. These data were supplemented by the analysis of archival radiological pictures of living individuals. The median stage for each age category was used to prepare the Atlas [17,18].

It should be noted that medical science is increasingly using innovations in the field of information technology, with particular emphasis on artificial intelligence methods, including neural modelling. These methods help to improve the effectiveness of treatment and accuracy of diagnosis of medical conditions [19,20].

The use of artificial neural networks in the processing of medical information and images helps to generalize the data contained even in X-rays with noise [21,22], thus enabling more efficient diagnostics and avoiding incorrect diagnoses [23]. Neural modelling can be the core of an expert system that assists the physician in the daily management of patient information, including the management of data during procedures, such as in the Da Vinci Robot [24,25].

Neural modelling is becoming increasingly popular in the biological and medical community [26]. It can be used in many diagnostic aspects like detection of pulmonary tumor in chest radiographs [27], in the classification of medical data [28], in the analysis of microscopic images [29]. Neural modeling has also been used in determining age and sex [30] and in clinical diagnostics related to endoscopy [31]. Neural modeling is also used in the determination of biomarkers [32] as well as in the detection of brain tumors [27,28,29,30,31,32,33]. Artificial intelligence methods are also used in the analysis of ECG-electrocardiogram-signal analysis [34,35], in Alzheimer’s and Parkinson’s diagnosis [36,37,38], urology [39], and oncology [40,41,42,43,44,45].

In dentistry, the Seok-Ki and Tae-Woo team [46] developed a neural model for the diagnosis of tooth extraction during orthodontic treatment. Neural models are also used in prosthetic [47] and conservative treatment [48]. In 2017, the Bunyarit team used the Demirijan method indicators in neural modelling to more accurately estimate the dental age of Malaysian children [49].

It should be noted that the early 2021 papers touch on age determination for children, adolescents, and adults using artificial neural networks. The paper by Mauer and team [50] presents the possibility of age estimation using a 3D image of the knee. However, even for the use of deep learning algorithms, the quality of the model is about 90% and the MAE error (mean absolute error) +/− is half a year. It should also be noted that this type of age determination is quite time consuming and expensive. The validity of using artificial neural network method is questioned [51]. From a study of a population of more than 3000 cases aged from 4 to 40 years, the method presented in this paper gives better results. However, it should be noted the fact of a large age range of the studied cases and the use of compiled indicators. Moreover, the method of convolutional networks differs from deep learning and traditional methods. Measurements on cephalometric images are of a different nature and concern not only teeth but also other bone parameters. Nearly 300 images were tested, but the results, although indicating a correlation, were not satisfactory.

The study of age in relation to tooth deterioration was conducted by [52]. In a study dating back to 2017, the team of researchers described a study on the use of CBCT (cone beam computed tomography) assessment of tooth destruction. In this study, the authors estimate age by determining the structural changes of the tooth. The CBCT technique used involves more expensive equipment. However, it should be emphasized that CBCT is a much more accurate examination, although it is not common in less developed countries. In contrast, the method itself does not use metrics to measure and automatic image analysis methods. The authors indicated a statistical R-value of 0.85.

The analysis of the literature in terms of the assessment of the chronological age, artificial intelligence methods, including the use of neural modelling methods, has shown that so far no highly effective and accurate methodology has been developed and published, which uses modern computer systems and tools to determine the chronological age on the basis of digital pantomographic images. Currently used analog methods are rather imprecise and subjective, which has created the necessity to search for new, more effective techniques and methods of determining the chronological age.

Therefore, work has been carried out in determining the features of pantomographic images that support the determination of metric age, and neural models were produced to support the process of identifying the age of children and adolescents. The whole conducted work was a new methodology of metric age assessment from pantomographic images.

## 2. Materials and Methods

### 2.1. Material

The source of the analyzed data was the database of patients of the University Centre of Dentistry and Specialist Medicine in Poznań, Poland. The research material consisted of 619 digital pantomographic images of children and adolescents aged 4 to 15 (from 48 to 144 months). At the age of 4 to 15, the development of teeth is best visible. The research group included 296 photos of girls and 323 photos of boys. All cases were verified and the photos that presented developmental abnormalities and disorders such as: systemic diseases, facial lesions, facial and dental malformations or dental hard tissues and pulp diseases were excluded. The Bioethics Committee of the Medical University of Poznań considered that the research carried out does not have the characteristics of a medical experiment and therefore agreed to carry out the relevant work.

### 2.2. Research Methodology

In order to carry out the work and to solve the scientific problem, actions were taken that included the following steps:

1. Acquisition of research material-pantomographic images of children and adolescents aged 4 to 15 (from 48 to 144 months);
2. Verification and exclusion of abnormal cases, e.g., with developmental disorders;
3. Preparation of a database of selected digital pantomographic images in MS Excel;
4. Determination of patients’ age at the moment of picture taking-expressed in months;
5. Determination of a set of tooth and bone parameters;
6. Collection of tooth and bone parameters using ImageJ software;
7. Combining the collected tooth and bone measurements with the patient base using MS Excel;
8. Definition of a set of indicators-values of proportions of measured tooth and bone parameters;
9. Processing of data;
10. Preparation of a teaching set for neural modelling;
11. Neural modelling in STATISTICA 7.1;
12. Verification of the produced models;
13. Determination of a set of indicators and parameters relevant for the assessment of chronological age;
14. Development of a methodology for the assessment of chronological age;

#### 2.2.1. Research Equipment and Software

The pantomographic photos used in the research process were taken with the Duerr Dental-VistaPano S Ceph camera. The standard camera of this type is equipped with an X-ray head with 0.5 mm focus and a digital sensor, Cls-CMOS matrix. In addition, this model of the camera enables shooting in S-PAN technology, which improves the sharpness and saturation of mineralized structures. The camera records digital images in DICOM 3.0 format, which is supported by DBSWIN [53]-specialized imaging software. The program supports 16-bit grayscale, as well as the creation, import and export of image data and databases. DBSWIN reproduces images dedicated to medicine, including oncology, cardiology, dentistry, ophthalmology, and surgery [53]. The measurements of tooth and bone parameters were performed in the free, open ImageJ 1.52a program [54]. In the course of research, a MS Excel 2007 spreadsheet was used to aggregate and structure the data obtained in the process of image processing and analysis, which also enables saving the data in *.csv format [55].

The process of generating a neural model was carried out using STATISTICA 7.1. software in the neural modeling module, which allows to create, validate, and test artificial neural network models. In this module it is also possible to perform a sensitivity analysis of variables of the developed models [56].

The neural modelling process was carried out in the STATISTICA 7.1. suite. The generation of neural models was performed in three stages, in the module “Neural Networks”. In the first stage the “Automatic designer” function was used. It tested 20 networks of each type; the program was to keep the top 10 networks of each type. The criterion for selecting the networks remembered for further analysis was to maintain the balance between the error and the diversity of networks. During this stage, PNN (Probabilistic Neural Network), GRNN (Generalized Regression Neural Network), RBF (Radial Basis Function), and three- and four-layer MLP (Multilayer Perceptron) networks were tested. After the analysis, it turned out that none of the produced models were characterized by favorable coefficients determining the quality of operation. The quality of the network is understood as the difference between the initial value of the network (calculated) and the set value (actual age). The higher the value for the training set is and the closer it is to 1, the higher the network quality [56]. However, it could be noted that among the whole set of networks produced, RBF-type networks showed some advantage. Therefore, in the next stage of modeling, the “Automatic designer” function was used to only generate networks of this type.

Automatic network designer is a feature of the STATISTICA package that allows you to define assumptions for selected, generated neural models. Owing to this function, the user can test models and select the optimal number of neurons for each topology. The program selects the values of the neural weights of individual networks and, depending on the settings, performs the task by selecting different networks with different topologies with information about their quality characteristics.

The remaining parameters of the program operation remained unchanged. However, also at this stage, the quality of performance of the generated networks was not satisfactory. Next, in the third stage of neural modeling, the “User network designer” function was used. Using this function, the type of network was determined: network performing generalized regression (RBF) and the number of hidden neurons, depending on the modelled number of input variables: 23 for the set containing cases of women and men, or 22 variables—for women and men separately. Entropy was assumed to be a function of the network classification error. Networks generated at this stage were subjected to an optimization-tuning, which consisted of neuronal reduction in the input layer of the network, reduction of hidden neurons, etc. 

Probabilistic Neural Network is one of the so-called Bayesian networks used to solve classification problems. PNNs use kernel approximation to estimate the likelihood function of learning classes. During network learning, it is important to determine the smoothing factor, which is user-defined and has a significant impact on the network learning process. This coefficient determines the degree of overlap in the sequence of learning instances fed to the network inputs. The probabilistic network returns an estimated probability value as a result. The advantage of this type of network is fast learning process, whereas the disadvantages include very complex structure.

Generalized Regression Neural Network resemble PNN type networks in their operation. They are used to solve only regression problems. These networks are characterized by a specific structure: the first layer of the network contains radial neurons, the second a weighted average of outputs from the previous layer, while the output layer has the task of normalizing the calculated values. This process consists in dividing the received signal values with the average calculated in the hidden layer and therefore the hidden layer contains one additional neuron.

Radial Basis Function networks are characterized by a single hidden layer containing radial neurons. The algorithm of these networks includes a Gaussian response surface, which is a strongly nonlinear function. Thus, using a single hidden layer, it is possible to model a function of arbitrary shape. To get the right result in RBF networks, linear neurons are used in the output layer with a linear activation function. RBF network learns much faster than MLP networks.

One of the most popular ANN (Artificial Neural Network) topologies is the Multi Layer Perceptron. Its concept was developed in 1986 by Rumelhart and McClelland. It consists of multiple layered neurons. Each computes a weighted sum of its inputs, which is the argument of the output value activation function. The multilayer perceptron is a one-way structure whose main advantage is that it can model functions of any complexity.

#### 2.2.2. Methodology for Obtaining Empirical Data

**Figure 1:**
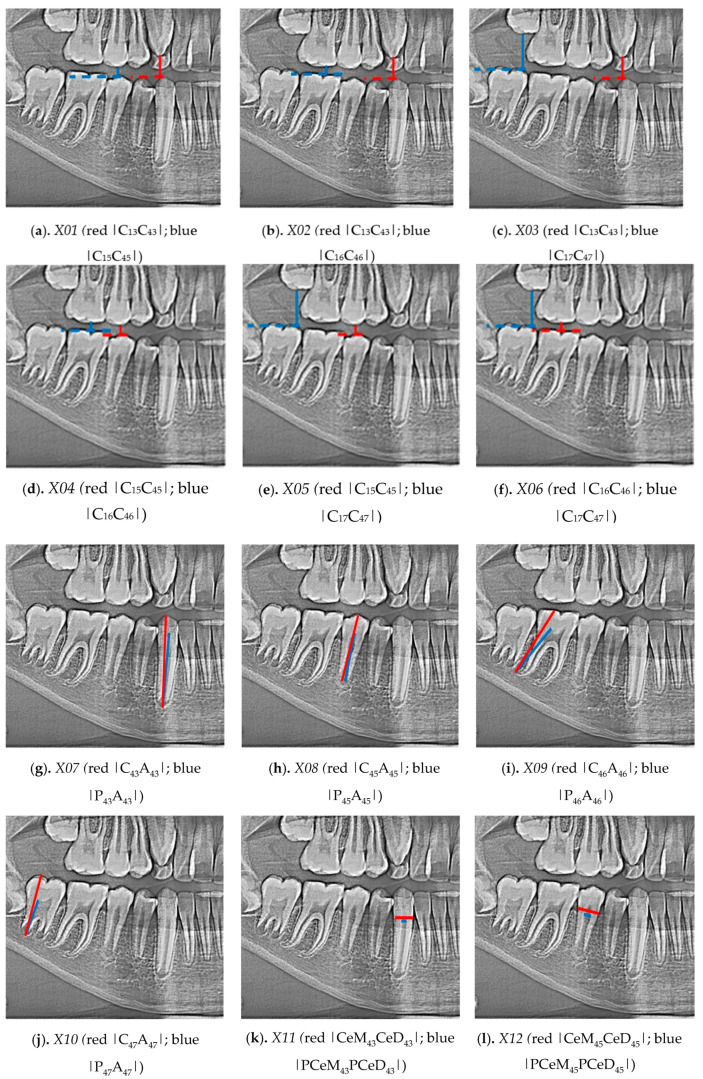

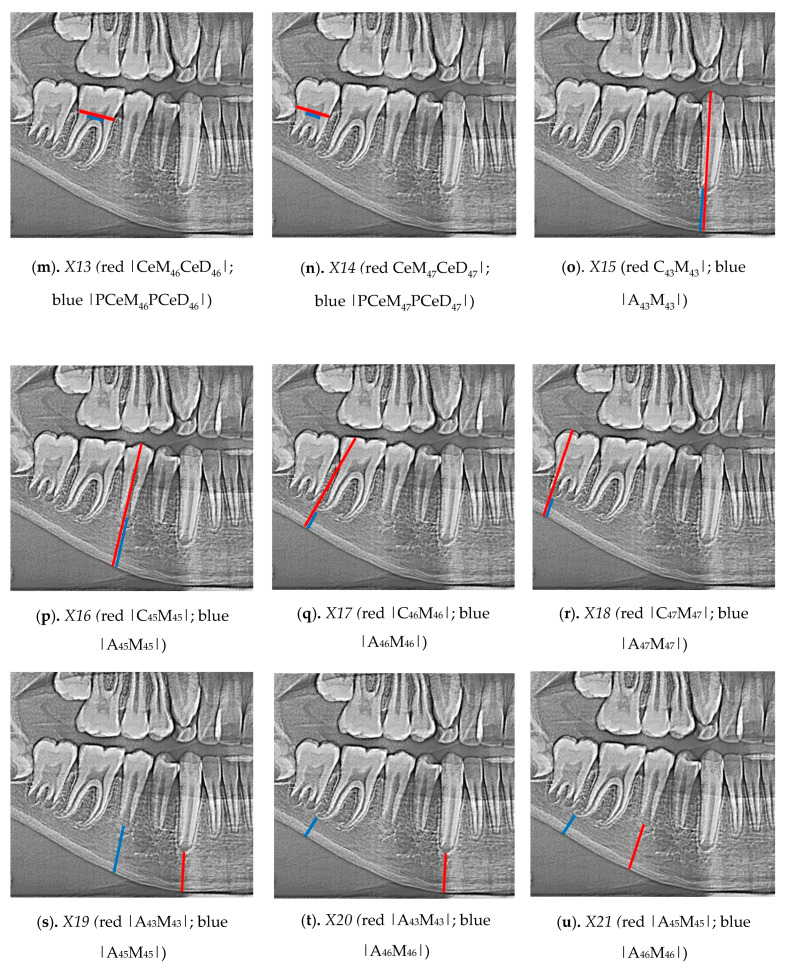
Developed set of 21 indicators was determined, i.e., distinctive tooth and bone parameters.

In the conducted research, an original, authored, and developed set of 21 indicators was determined, i.e., distinctive tooth and bone parameters, which were developed in the form of mathematical proportions:

1. Indicator X01 (Figure 1a) ratio between section |C13C43| and section C15C45|
2. Indicator X02 (Figure 1b) ratio between section |C13C43| and section |C16C46|
3. Indicator X03 (Figure 1c) ratio between section |C13C43| and section |C17C47|
4. Indicator X04 (Figure 1d) ratio between section |C15C45| and section |C16C46|
5. Indicator X05 (Figure 1e) ratio between section |C15C45| and section |C17C47|
6. Indicator X06 (Figure 1f) ratio between section |C16C46| and section |C17C47|
7. Indicator X07 (Figure 1g) ratio between section |C43A43| and section |P43A43|
8. Indicator X08 (Figure 1h) ratio between section |C45A45| and section |P45A45|
9. Indicator X09 (Figure 1i) ratio between section |C46A46| and section |P46A46|
10. Indicator X10 (Figure 1j) ratio between section |C47A47| and section |P47A47|
11. Indicator X11 (Figure 1k) ratio between section |CeM43CeD43| and section |PCeM43PCeD43|
12. Indicator X12 (Figure 1l) ratio between section |CeM45CeD45| and section |PCeM45PCeD45|
13. Indicator X13 (Figure 1m) ratio between section |CeM46CeD46| and section |PCeM46PCeD46|
14. Indicator X14 (Figure 1n) ratio between section |CeM47CeD47| and section |PCeM47PCeD47|
15. Indicator X15 (Figure 1o) ratio between section |C43M43| and section |A43M43|
16. Indicator X16 (Figure 1p) ratio between section |C45M45| and section |A45M45|
17. Indicator X17 (Figure 1q) ratio between section |C46M46| and section |A46M46|
18. Indicator X18 (Figure 1r) ratio between section |C47M47| and section |A47M47|
19. Indicator X19 (Figure 1s) ratio between section |A43M43| and section |A45M45|
20. Indicator X20 (Figure 1t) ratio between section |A43M43| and section |A46M46|
21. Indicator X21 (Figure 1u) ratio between section |A45M45| and section |A46M46|

The measurements were made manually with using ImageJ software.

## 3. Results

Various network topologies (PNN, GRNN, RBF, and MLP) were tested during the research. RBF networks were characterized by the best quality indicators, therefore the three best models determining the chronological age were obtained: 1. women and men, 2. women, 3. men. The results of the research are three neural models that determine the chronological age. Three possibilities were tested on different groups to identify which model is the most accurate and to determine which variables are characteristic of gender groups and which variables affect the accuracy of the modelling result.

In the process of neural modelling for 619 cases of women and men, 23 input variables were involved (X01-X21, gender and age expressed in months). The output variable was “MONTHS”, the remaining variables were input variables.

The sex “SEX” was a categorized variable. Months are a continuous variable, defining the number of months from birth to the date of the study. Variables from X01 to X21 were continuous in nature and marked the calculated indicators characterized above.

In the process of neural modelling for 296 female cases 22 variables were involved (the gender variable was eliminated). The output variable was “MONTHS”, the other variables were input variables.

In the process of neural modelling for 323 cases of men 22 variables were involved (the gender variable was eliminated). The output variable was “MONTHS”, the remaining variables were input variables.

The division into learning, validation, and testing set was carried out at random for each data collection. The learning set takes part in the process of generating the network, the validation set takes part in tuning the model, while the test set is excluded from the process of neural modelling—these are raw, previously unused data to check and evaluate the artificial neural network. As a standard, the data set is divided into a 2:1:1 ratio (50%, 25%, 25%).

**Figure 2:**
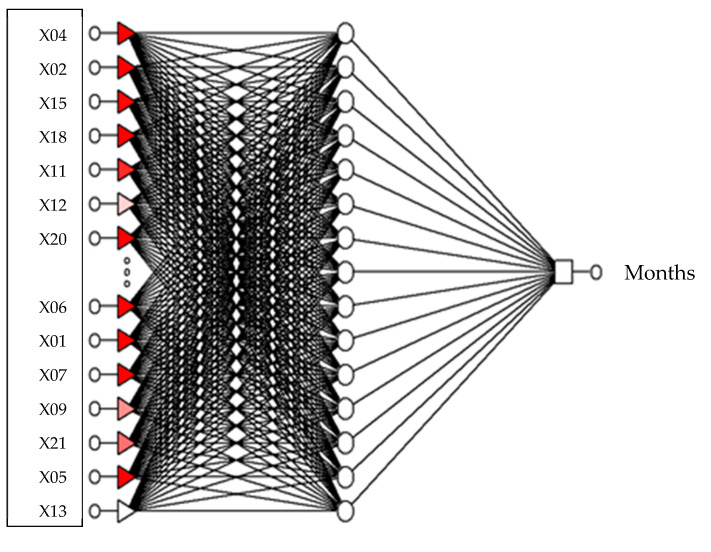
The produced model RBF 22:22-15-1:1, which determines the chronological age of women and men.

Neural model RBF 22:22-22-1:1 (Figure 2) (Table 1), determining the chronological age for women and men. The network sensitivity analysis is shown in Table 2.

**Figure 3:**
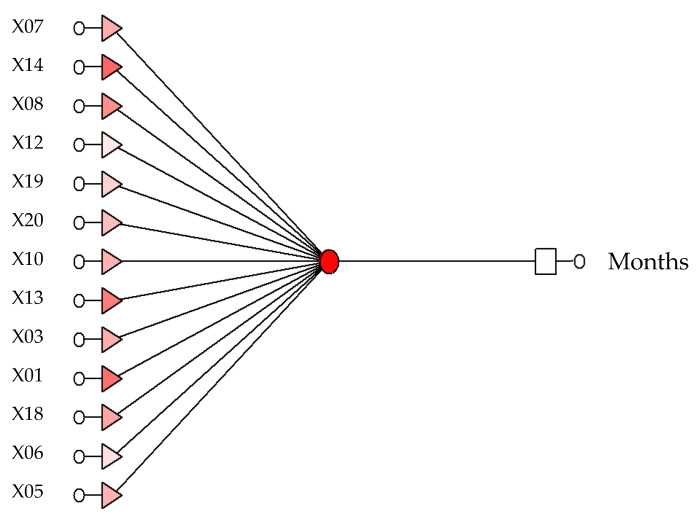
The produced model RBF 13:13-1-1:1, which determines the chronological age of women and men.

Neural model RBF 13:13-1-1:1 (Figure 3) (Table 3), determining the chronological age for women. The network sensitivity analysis is shown in Table 4.

**Figure 4:**
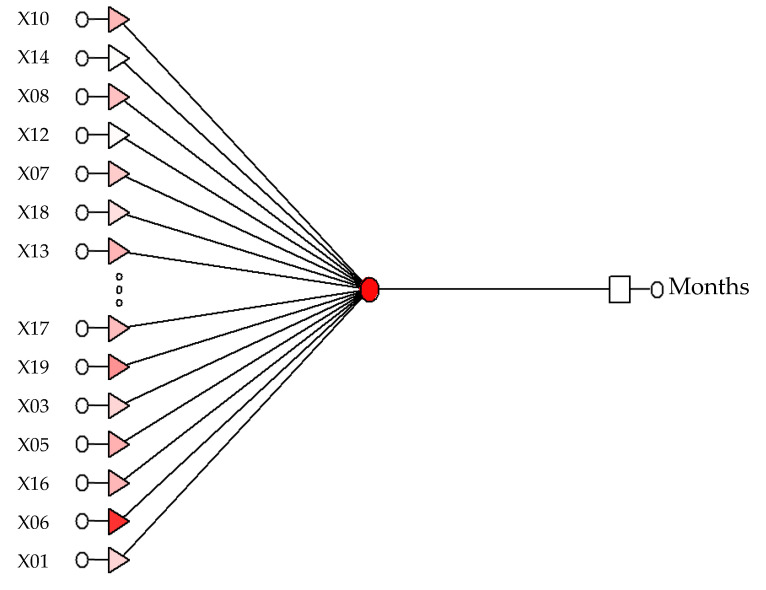
The produced model RBF 18:18-1-1:1 that determines the chronological age of women and men.

Neural model RBF 18:18-1-1:1 (Figure 4) (Table 5) determining the chronological age for men. The network sensitivity analysis is shown in Table 6.

## 4. Discussion

The result of the conducted research is the development of a new, original methodology for determining the chronological age of children between the ages of 4 and 15 (from 48 to 144 months). The paper identifies and presents an original set of 21 tooth and bone parameters, indicators, owing to which, on the basis of digital pantomographic images, using neural modelling methods, it is possible to accurately and effectively assess the chronological age. The accuracy of the proposed method of chronological age assessment is 99.7%.

During the study, three optimal neural models were developed to determine the chronological age: female and male RBF 22:22-15-1:1 with test set quality of 0.9974 and test set error of 0.0365, female only RBF 13:13-1-1:1 with test set quality of 0.9631 and test set error of 0.0336, and male only RBF 18:18-1-1:1 with test set quality of 0.9993, and test set error of 0.0398. The measure of error is RMSE (Root Mean Square Error). A summary of the models is presented in the Table 7.

To work properly, each model needs appropriate input variables and indicators that have been defined in the sensitivity analysis process. For the model RBF 22:22-15-1:1 these are the indicators: X04; X02; X15; X18; X11; X12; X20; X14; X03; X16; X08; X19; X17; X10; SEX; X06; X01; X07; X09; X21; X05; X13. For model RBF 13:13-1-1:1, these are the indicators: X07; X14; X08; X12; X19; X20; X10; X13; X03; X01; X18; X06; X05. For the model RBF 18:18-1-1:1, these are the indicators: X18; X20; X16; X10; X03; X19; X14; X01; X07; X09; X05; X06; X17; X13; X08; PLEC; X12; X11.

Variables relevant for each of the analyzed models are X01; X03; X05; X06; X07; X08; X10; X12; X13; X14; X18; X19. A detailed analysis of the models produced is shown in Table 8.

The newly developed methodology is based on: taking a digital pantomographic picture; making measurements of fixed sections on digital pantomographic pictures (measurements with ImageJ software), exporting a set of these measurements to a spreadsheet, e.g., MS Excel, and calculating the original indicators-proportions-proposed in the course of the conducted tests; subsequently, the calculated values should be transformed into a *.csv file and entered into the neural model used to determine the chronological age.

The novelty of the developed method lies in the use of a pantomogarphy photograph and the use of a proprietary set of developed indicators. Currently, there is no scientific or commercial method that allows to estimate metric age in this way, using neural modeling methods.

Comparing the conducted study with other works [46,47,48,49], it should be emphasized that the works performed were dedicated to children of a certain age. The teaching set included a representative research group, which probably influenced the neural modeling process. In comparison with other cited methods, the assessment of metric age operates on two-dimensional pantomographic images. The set of indicators used to describe the image is the author’s. It was developed as a result of the clinical experience of the research team and forms the basis of the work carried out.

A limitation in the application of the developed method and technique is the use of 2D images (although it should be noted that taking a pantomographic image is inexpensive and common in clinical practice). It should also be emphasized that the models produced are dedicated to children and adolescents aged 48 to 144 months. The presented method is simpler than others cited in this work.

Mention should be made of the research of the Bunyarit [57] team, who used the artificial neural network method to estimate the age of children and adolescents aged 5 to 18 years. The accuracy of their model based on Chaillet and Demirjian’s 8-tooth method for dental age estimation was 0.938 to 0.951 (fit coefficient value).

The paper by Galibourg and team [58] uses the use of deep learning methods and Demirjian’s method to estimate the dental age. The authors report that the study was conducted on a group of 3605 images of people aged 2 to 24 years. The MAE error for their method was 0.811 years, which was more accurate than methods previously used.

In Pan and team’s work [59], a modified method of Demirjan and Willems was presented, which increased the accuracy of metric age estimation to about 83%. The study was conducted on a population of 2367 Chinese children aged 5 to 16 years. Traditional statistical methods were used to process the results.

Wallraff’s team [60] obtained promising results from an analysis of pantomographic images of 14,000 patients aged 11 to 20 years. The error of the developed model was more than 17%, but deep learning methods and convolutional networks seem to be an ideal tool in solving this type of issues.

Most recent work on metric age assessment is based on traditional, atlas-based methods [61,62]. There are also works on adult age assessment [63,64,65]. It seems that the challenge of the 21st century is to develop modern tools that would allow measurements and classifications to be made automatically. Therefore, it is reasonable to develop neural image analysis methods in medical imaging problems.

The next stage of metric age estimation work should be related to the application of deep learning methods or using convolutional networks.

## 5. Conclusions

This newly developed methodology may serve as an algorithm for implementation in a computer application, which will automatically determine the chronological age of children and adolescents between the ages of 4 and 15 (from 48 to 144 months). The conducted research allowed to formulate the following conclusions:

1. It is possible to develop a new methodology for the assessment of chronological age on the basis of digital pantomographic images with the determination of a new set of tooth and bone parameters.
2. It is possible to develop a neural model, which will facilitate the assessment of chronological age.
3. The optimal model for assessing the chronological age of women and men is RBF 22:22-15-1:1 with 99% test set quality and 3.7% RMSE set error.
4. The optimal model for assessing the metric age of women is RBF 13:13-1-1:1 with 96% test set quality and 3.4% RMSE set error.
5. The optimal model for assessing the metric age of men is RBF 18:18-1-1:1 with 99% test set quality and 4.0.% RMSE set error.
6. This newly developed methodology is an effective and innovative tool in the assessment of the chronological age of Polish children and adolescents aged between the ages of 4 and 15 (from 48 to 144 months).

The developed model works only for pantomographic images of children and adolescents aged 4 to 15 years. The models are dedicated to this age group and to the developed indicators. On the other hand, the measurements obtained during the study are dimensionless—they have a charter of proportions which allows universalizing the use of images from different pantomographic cameras.

The next recommended step in the research will be to use deep learning methods and compare the network characteristics with the new models, and to conduct a sensitivity analysis of the variables needed to determine metric age using deep learning methods.

## Figures and Tables

**Table 1:** Characteristics of the RBF 22:22-15-1:1.

RBF 22:22-15-1:1	
Training quality	0.9738
Validation quality	0.9981
Testing quality	0.9973
Training error	0.0359
Validation error	0.0373
Testing error	0.0365

**Table 2:** Sensitivity analysis of the RBF 22:22-15-1:1 network.

Variable	The Quotient	Rank
X04	1.0126	1
X02	1.0125	2
X15	1.0122	3
X18	1.0057	4
X11	1.0051	5
X12	1.0051	6
X20	1.0024	7
X14	1.0010	8
X03	1.0010	9
X16	1.0010	10
X08	1.0009	11
X19	1.0008	12
X17	1.0004	13
X10	1.0004	14
SEX	1.0003	15
X06	1.0002	16
X01	1.0002	17
X07	1.0001	18
X09	1.0001	19
X21	1.0001	20
X05	1.0000	21
X13	0.9999	22

**Table 3:** Characteristics of the RBF 13:13-1-1:1.

RBF 13:13-1-1:1	
Training quality	0.9945
Validation quality	0.9740
Testing quality	0.9631
Training error	0.0366
Validation error	0.0376
Testing error	0.0336

**Table 4:** Sensitivity analysis of the RBF 13:13-1-1:1 network.

Variable	The Quotient	Rank
X07	1.0126	1
X14	1.0055	2
X08	1.0044	3
X12	1.0025	4
X19	1.0007	5
X20	0.9999	6
X10	0.9986	7
X13	0.9982	8
X03	0.9965	9
X01	0.9956	10
X18	0.9956	11
X06	0.9913	12
X05	0.9894	13

**Table 5:** Characteristics of the RBF 18:18-1-1:1.

RBF 18:18-1-1:1	
Training quality	0.9997
Validation quality	0.9989
Testing quality	0.9993
Training error	0.0380
Validation error	0.0417
Testing error	0.0398

**Table 6:** Sensitivity analysis of the RBF 18:18-1-1:1 model.

Variable	The Quotient	Rank
X10	1.0544	1
X14	1.0354	2
X08	1.0254	3
X12	1.0216	4
X07	1.0214	5
X18	1.0162	6
X13	1.0127	7
X15	1.0094	8
X11	1.0037	9
X04	1.0007	10
X02	1.0007	11
X17	0.9958	12
X19	0.9934	13
X03	0.9900	14
X05	0.9821	15
X16	0.9806	16
X06	0.9253	17
X01	0.9193	18

**Table 7:** The summary of the generated models.

Name of the Learning Set	Women and Men	Women	Men
**Network type**	**RBF 22:22-15-1:1**	**RBF 13:13-1-1:1**	**RBF 18:18-1-1:1**
**Training quality**	0.9738	0.9945	0.9997
**Validation quality**	0.9981	0.9740	0.9989
**Testing quality**	0.9974	0.9631	0.9993
**Training error**	0.0359	0.0366	0.0380
**Validation error**	0.0374	0.0376	0.0417
**Testing error**	0.0365	0.0336	0.0398

**Table 8:** The sensitivity analysis of RBF 22:22-15-1:1, RBF 13:13-1-1:1, and RBF 18:18-1-1:1 models.

Network Type	RBF 22:22-15-1:1		RBF 13:13-1-1:1		RBF 18:18-1-1:1	
Variable	The Quotient	Rank	The Quotient	Rank	The Quotient	Rank
X01	1.0002	17	0.9956	10	0.9193	18
X02	1.0125	2			1.0007	11
X03	1.0010	9	0.9965	9	0.9900	14
X04	1.0126	1			1.0007	10
X05	0.9999	21	0.9894	13	0.9821	15
X06	1.0002	16	0.9913	12	0.9253	17
X07	1.0001	18	1.0126	1	1.0214	5
X08	1.0009	11	1.0044	3	1.0254	3
X09	1.0001	19				
X10	1.0004	14	0.9986	7	1.0544	1
X11	1.0051	5			1.0037	9
X12	1.0051	6	1.0026	4	1.0216	4
X13	0.9999	22	0.9982	8	1.0127	7
X14	1.0010	8	1.0055	2	1.0354	2
X15	1.0122	3			1.0094	8
X16	1.00010	10			0.9806	16
X17	1.0004	13			0.9958	12
X18	1.0057	4	0.9956	11	1.0161	6
X19	1.0008	12	1.0008	5	0.9934	13
X20	1.0024	7	0.9999	6		
X21	1.0001	20				
X22						
SEX	1.0003	15				

## Abbreviations

A43	apex of the root of the tooth 43
A45	apex of the root of the tooth 45
A46	apex of the distal root of the tooth 46
A47	apex of the distal root of the tooth 47
C13	top of the crown of the tooth 13
C15	top of the cheek nodule of the tooth 15
C16	top of the distal cheek nodule of the tooth 16
C17	top of the distal cheek nodule of the tooth 17
C43	top of the crown of the tooth 43
C45	top of the cheek nodule of the tooth 45
C46	top of the distal cheek nodule of the tooth 46
C47	top of the distal cheek nodule of the tooth 47
CeD43	distal cervical point of the tooth 43
CeD45	distal cervical point of the tooth 45
CeD46	distal cervical point of the tooth 46
CeD47	distal cervical point of the tooth 47
CeM43	mesial cervical point of the tooth 43
CeM45	mesial cervical point of the tooth 45
CeM46	mesial cervical point of the tooth 46
CeM47	mesial cervical point of the tooth 47
CM16	top of the mesial cheek nodule of the tooth 16
CM17	top of the mesial cheek nodule of the tooth 17
CM46	top of the mesial cheek nodule of the tooth 46
CM47	top of the mesial cheek nodule of the tooth 47
M43	a point on the lower edge of the mandible in the projection of a straight line through points C43 and A43
M45	a point on the lower edge of the mandible in the projection of a straight line through points C45 and A45
M46	point on the lower edge of the mandible in the projection of a straight line through points C46 and A46
M47	a point on the lower edge of the mandible in the projection of a straight line through points C47 and A47
P43	upper point of the pulp chamber of the tooth 43
P45	upper point of the pulp chamber of the tooth 45
P46	top of distal corner of the pulp chamber of the tooth 46
P47	top of distal corner of the pulp chamber of the tooth 47
PCeD43	distal point of the pulp chamber of the tooth 43 in the cervical area
PCeD45	distal point of the pulp chamber of the tooth 45 in the cervical area
PCeD46	distal point of the pulp chamber of the tooth 46 in the cervical area
PCeD47	distal point of the pulp chamber of the tooth 47 in the cervical area
PCeM43	mesial point of the pulp chamber of the tooth 43 in the cervical area
PCeM45	mesial point of the pulp chamber of the tooth 45 in the cervical area
PCeM46	mesial point of the pulp chamber of the tooth 46 in the cervical area
PCeM47	mesial point of the pulp chamber of the tooth 47 in the cervical area
